# Reappraisal of the management of Vogt–Koyanagi–Harada disease: sunset glow fundus is no more a fatality

**DOI:** 10.1007/s10792-016-0395-0

**Published:** 2016-11-14

**Authors:** Carl P. Herbort Jr, Ahmed M. Abu El Asrar, Joyce H. Yamamoto, Carlos E. Pavésio, Vishali Gupta, Moncef Khairallah, Ilknur Tugal-Tutkun, Masoud Soheilian, Masuru Takeuchi, Marina Papadia

**Affiliations:** 1 Retinal and Inflammatory Eye Diseases, Centre for Ophthalmic Specialized Care (COS), Clinic Montchoisi Teaching Centre, Rue Charles-Monnard 6, 1003 Lausanne, Switzerland; 2 Department of Ophthalmology, University of Lausanne, Lausanne, Switzerland; 3 Department of Ophthalmology, Dr. Nasser Al-Rashid Research Chair in Ophthalmology, College of Medicine, King Saud University, Riyadh, Saudi Arabia; 4 Department of Ophthalmology, University of Sao Paulo School of Medicine, Sao Paulo, Brazil; 5 National Institute for Health Research, Biomedical Research Centre at Moorfields Eye Hospital, NHS Foundation Trust, UCL Institute of Ophthalmology, London, UK; 6 Department of Ophthalmology, Post-graduate Institute, Chandigarh, India; 7 Department of Ophthalmology, Fattouma Bourghiba University Hospital, Faculty of Medicine, University of Monastir, Monastir, Tunisia; 8 Department of Ophthalmology, Istanbul Faculty of Medicine, Istanbul University, Istanbul, Turkey; 9 Department of Ophthalmology and Ophthalmic Research Centre, Labbafinejad Medical Centre, Shahid Beheshti Medical University, Teheran, Iran; 10 Department of Ophthalmology, National Defence Medical College, Tokorozawa, Saitama Japan; 11 Department of Ophthalmology, Ospedale Padre Antero Micone, Genoa, Italy

**Keywords:** Vogt–Koyanagi–Harada disease, Stromal choroiditis, autoimmune disease, Granulomatous uveitis, Indocyanine green angiography, Immunosuppressive therapy

## Abstract

**Purpose:**

Vogt–Koyanagi–Harada (VKH) disease is a primary autoimmune stromal choroiditis. Aim of the study was to gather a body of evidence from the literature and from experts that systemic corticosteroid combined with non-steroidal immunosuppressive therapy should become the standard of care in initial-onset VKH disease.

**Methods:**

Literature was reviewed and leading experts in VKH were consulted in different parts of the world in order to put forward a consensus attitude in the management of initial-onset VKH disease.

**Results:**

There was a substantial body of evidence in the literature that early aggressive and sustained corticosteroid and non-steroidal immunosuppressive therapy in initial-onset VKH disease allows to achieve full control of choroidal inflammation, eliminating any subclinical choroidal inflammation, and substantially reduces recurrences with improvement of anatomical and functional outcomes. This was in agreement with experts’ opinion and practice. ICGA was the method of choice to monitor disease evolution.

**Conclusion:**

Since the choroidal space is easily accessible to systemic therapy and because inflammation in VKH disease is exclusively originating from the choroidal stroma, early and sustained treatment right at the onset of the disease process with dual corticosteroid and non-steroidal immunosuppressive therapy can result in full “healing” in many cases preventing sunset glow fundus which results from depigmentation from chronic uncontrolled inflammation.

## Introduction

Vogt–Koyanagi–Harada (VKH) disease is a bilateral granulomatous panuveitis with exudative retinal detachments [1] found to be caused by an autoimmune reaction against stromal melanocyte-associated proteins [2–5]. In the acute phase, ocular disease is classically associated with inflammation of the meninges (cerebrospinal fluid mononuclear pleocytosis) and dysacusis [6, 7]. The initial-onset acute disease typically exhibits granulomatous choroiditis with exudative retinal detachment and optic disc hyperemia and swelling, subsequently involving the anterior segment and finally developing into chronic recurrent granulomatous anterior uveitis if not properly treated, with typical “sunset glow fundus” (SGF) [8–10], as well as persisting meningitis, persisting auditory disturbances as well as integumentary changes (vitiligo, alopecia and poliosis) [11].

## Knowing the disease mechanism is the key to its management: VKH is a primary stromal choroiditis

**Fig. 1:**
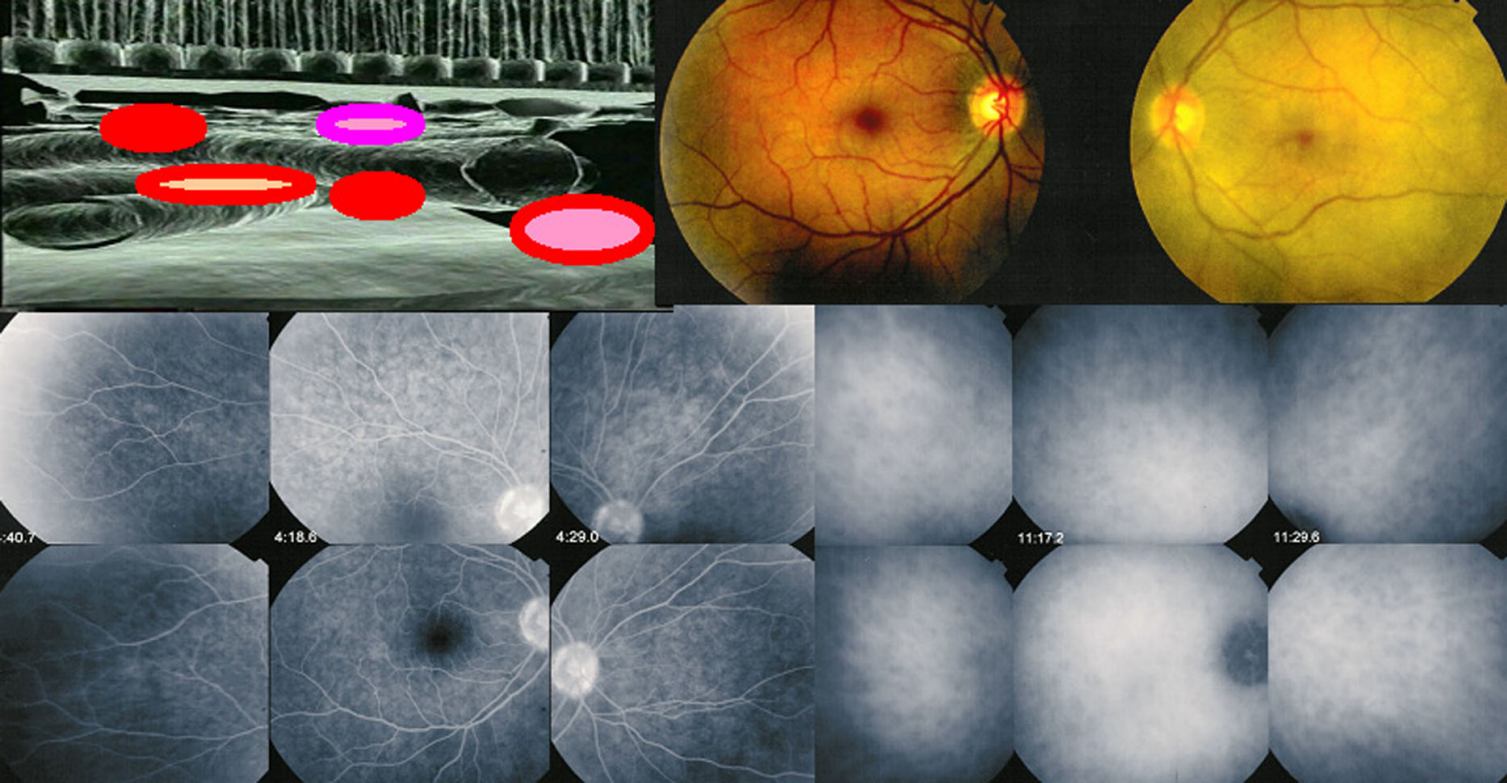
During the prodromal stage of the disease, subclinical choroidal inflammation is silently developing in the choroidal stroma as shown on cartoon (*top left*). This subclinical choroidal involvement can only be detected by ICGA, possibly by choroidal OCT. On the fundus pictures shown on top right, the right fundus is discoloured yellow due to massive choroidal infiltration. The right fundus looks normal and this patient was diagnosed as “unilateral” VKH disease. FA shows no lesions (*six bottom left frames*) but ICGA (*six bottom right frames*) clearly shows numerous hypofluorescent *dark dots* (HDDs) indicating choroidal granulomas in the apparently uninvolved eye

At the start of disease, patients may complain of headaches (a precious diagnostic element very often present), nausea, vertigo, fever, meningismus scalp hypersensitivity and orbital pain [12, 13]. This first, prodromal stage of disease is usually lasting for a few days/weeks and corresponds at the ocular level to subclinical inflammation starting exclusively in the choroidal stroma, without any other ocular clinical sign to indicate the presence of the inflammatory process, which can only be identified at this stage by performing indocyanine green angiography (ICGA) [14]. (Fig. 1).

**Fig. 2:**
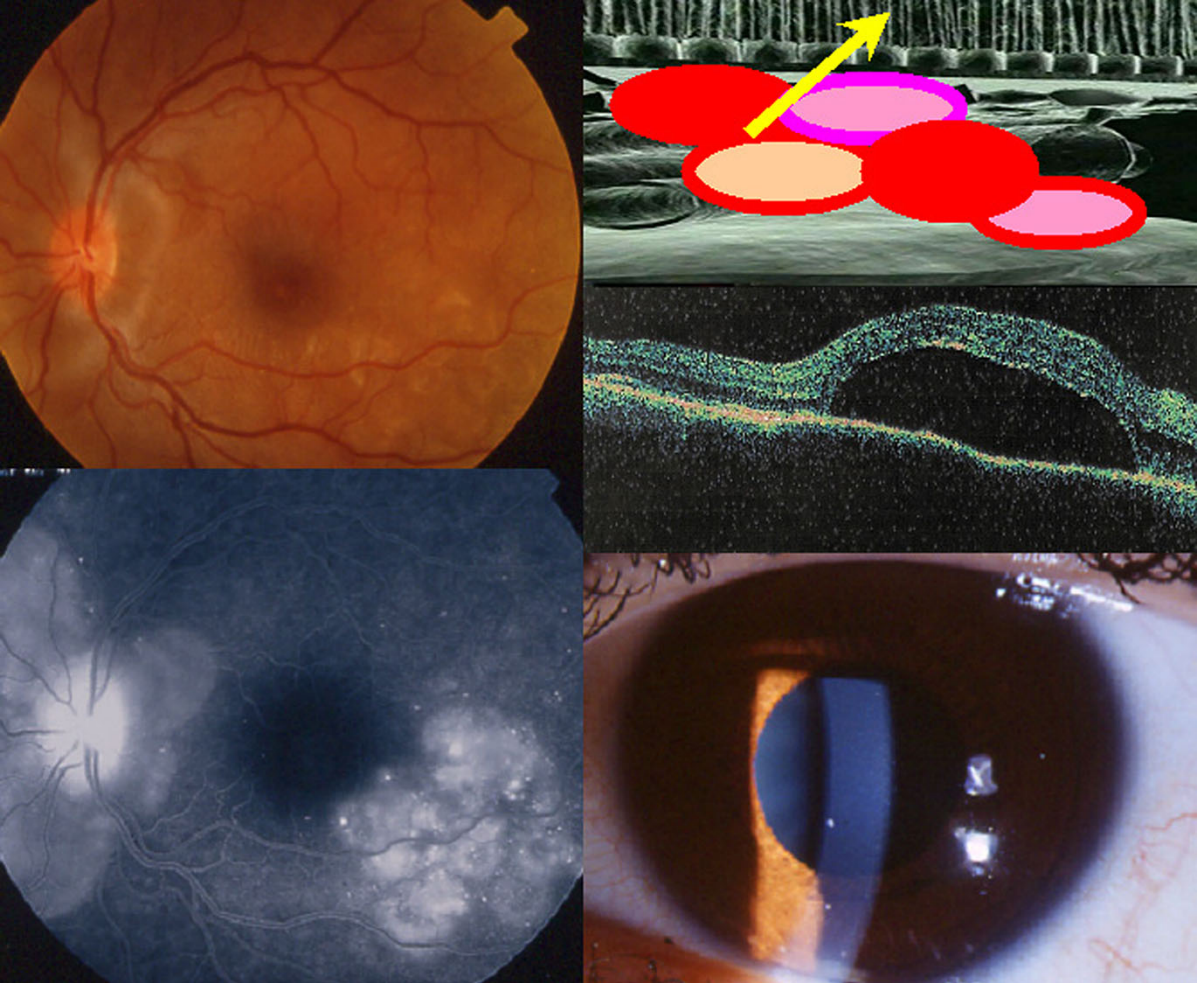
Acute exudative initial-onset VKH disease. Peripapillary and retinal exudative detachments seen on fundoscopy (*top left*) and FA (*bottom left*). Extrachoroidal structures are involved when choroidal disease spills over to neighbouring structures (cartoon, *top right*). Optical coherence tomography (OCT) shows clearly the detachment of the neurosensory retina (middle figure on the *right*). Moderate granulomatous anterior uveitis accompanying posterior involvement. (*bottom right*)

Subsequently, the disease becomes clinically apparent when choroidal inflammation spills over, usually in a cataclysmic fashion into the neighbouring structures including the optic disc, the retina, the ciliary body and sometimes also the anterior chamber. The expression of the disease in this second, uveitic/exudative stage of the disease, is that of a bilateral mostly granulomatous panuveitis with predominantly posterior involvement [9, 10, 15]. Classical clinical signs are bilateral papillitis, bilateral serous detachments of the retina, limited to moderate vitritis and sometimes an anterior usually granulomatous uveitis with minimal to moderate anterior chamber flare and cells associated with small to mid-size granulomatous keratic precipitates (KPs) [9, 10, 15]. (Fig. 2) At disease onset, the anterior uveitis can initially be non-granulomatous. Sometimes the degree of intraocular inflammation is such, in early acute disease, that there is supraciliary fluid accumulation at the origin of a ciliary detachment causing myopisation and sometimes angle-closure glaucoma [15].

In initial-onset acute disease, the most severe involvement occurs posteriorly. It is of utmost importance to be aware of the fact that the initial inflammatory event is taking place in the uvea starting in the choroid and more precisely in the choroidal stroma, the primary origin of all inflammation, hence the name of primary stromal choroiditis [16]. The other structures such as the retina and optic disc involved during the acute, clinically apparent phase of disease, are only involved secondarily to the choroidal inflammatory reaction. Inflammation in these structures is merely the consequence of the choroiditis; they do not generate inflammation by themselves [17].

Therefore, the first element that should determine our approach to the management of VKH disease is the fact that the single and exclusive source of all intraocular inflammation comes from the choroidal stroma. This makes VKH disease unique when compared to other choroiditis entities such as sarcoidosis which involves both choroid and retina at random [18] or birdshot retinochoroiditis where there is dual, parallel, independent choroidal and retinal involvement [19]. Hence management in VKH disease should be principally aimed at the choroidal inflammation with subsequent resolution of all intraocular inflammation. The choroid being a highly vascularized structure is very accessible to systemic treatment, and there is no rationale for intravitreal therapy which, as expected, has been shown not to be very efficient [20].

Isolated cases advocating intraocular corticosteroids have been published but do not represent a serious argument, one of these cases not even being a VKH case [21].

## The essential role of indocyanine green angiography (ICGA) in the appraisal and management of VKH (Figs. 3, 4, 5)

As the initial inflammatory events start in the choroidal stroma, ICGA is indeed the method of choice to establish and monitor choroidal inflammation [22, 23]. In that respect, ICGA is able to detect early subclinical disease as well as subclinical recurrences during tapering of therapy [24]. ICGA was also able to show occult concomitant choroidal inflammation during anterior segment recurrence when there seemingly was no posterior uveal activity [25].

**Fig. 3:**
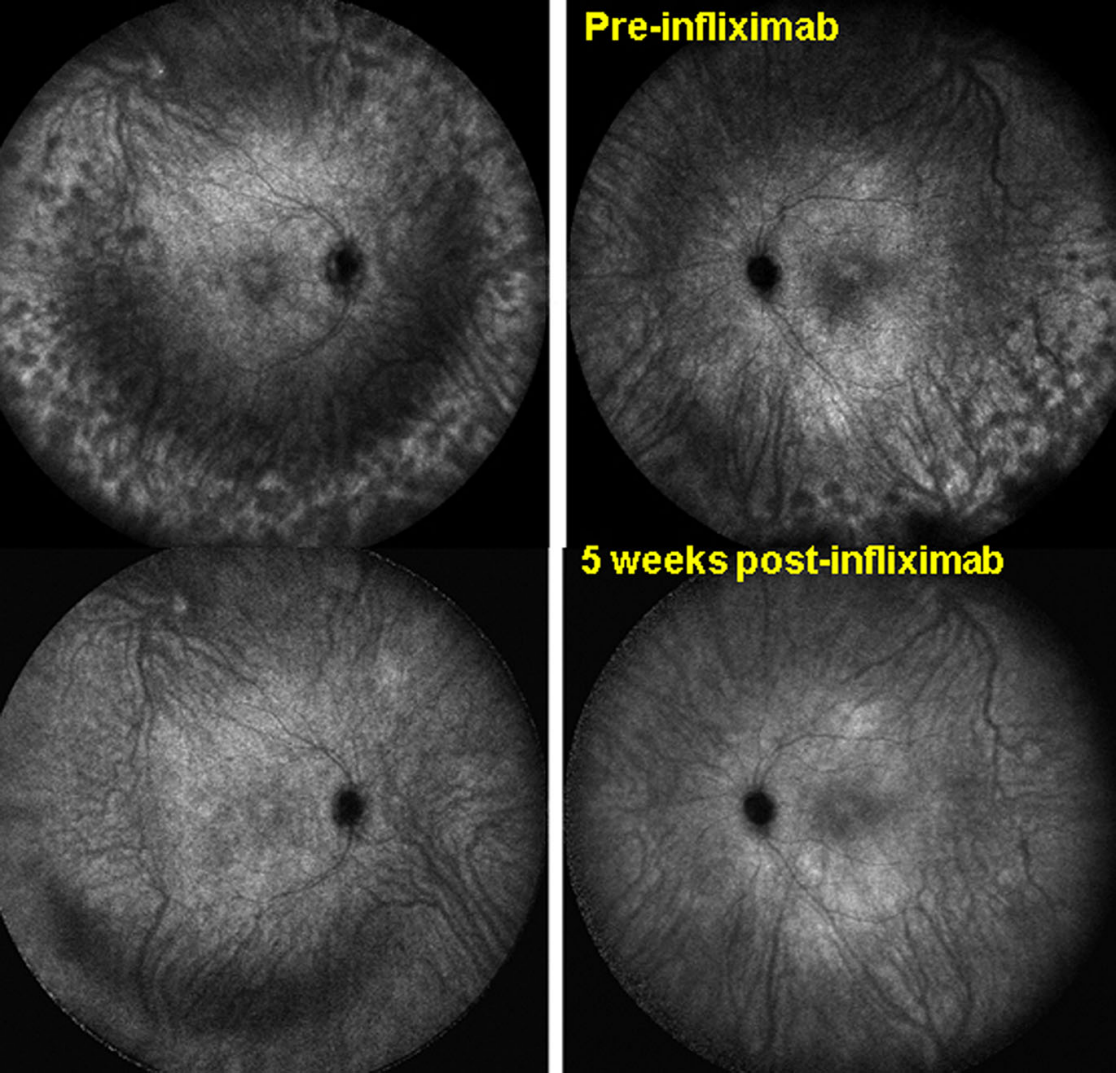
Sensitivity and global panfundal information given by ICGA. Case of VKH disease responsive to initial highly dosed corticosteroids with peripheral recurrence under mycophenolic acid (Myfortic ^®^) and cyclosporine (CsA) characterized by numerous HDDs (*top pictures*). After introduction of infliximab complete resolution of choroiditis within 5 weeks (*bottom pictures*), establishing infliximab as the therapy to which this patient was responsive. Posterior pole involvement was minimal and choroidal OCT did not reflect spectacular improvement of choroiditis

**Fig. 4:**
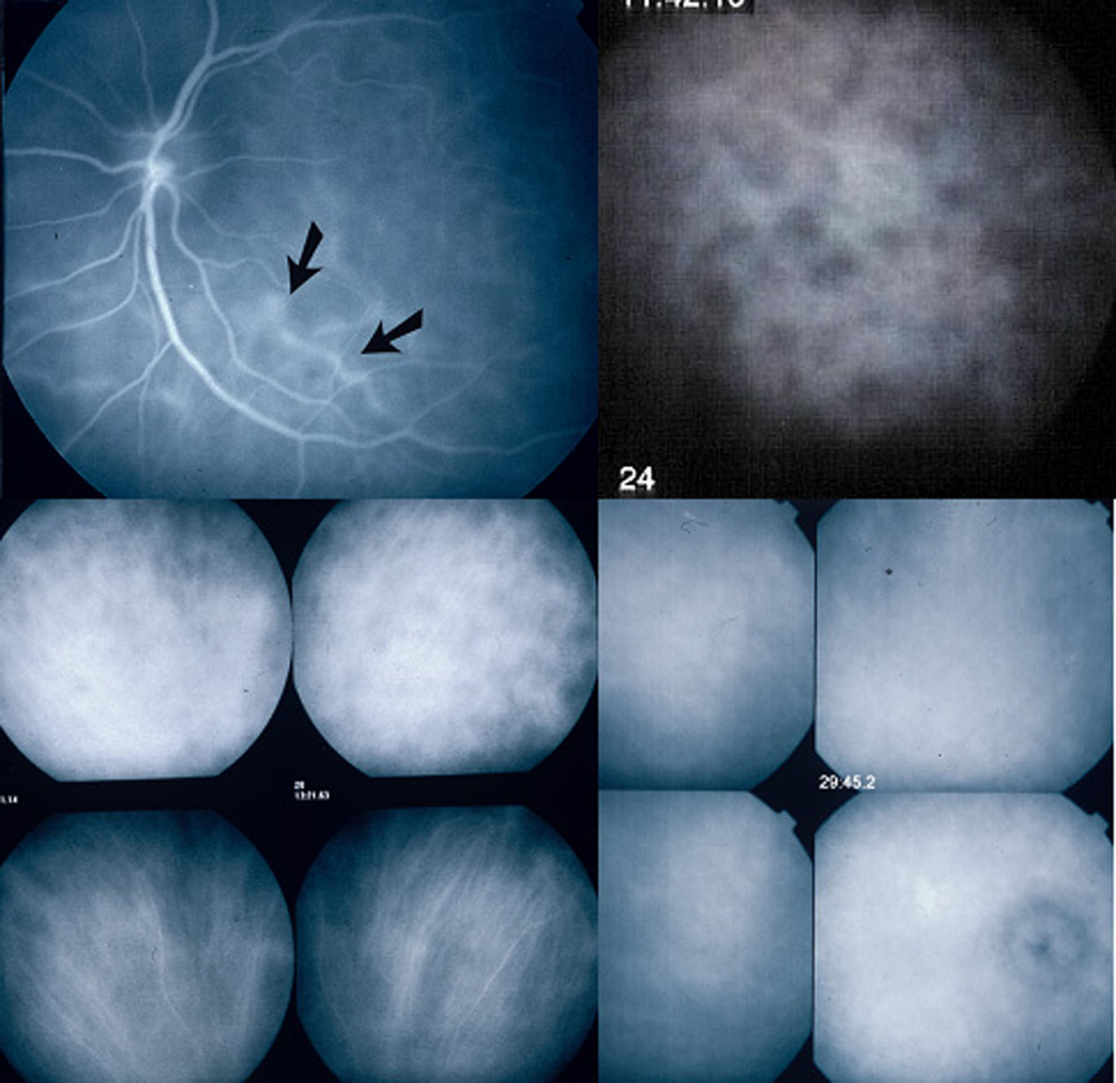
Indocyanine green angiographic signs. ICGA is the only technique to analyse choroidal inflammatory signs including early stromal hyperfluorescent vessels (*top left*), hypofluorescent *dark dots* (HDD) indicating choroidal granuloma (*top right*), fuzzy indistinct choroidal vessels (top 2 frames of *bottom left* quartett). After 3 days of intravenous 1000 mg daily methylprednisolone the normal pattern of vessels is again recognizable (bottom 2 frames of *bottom left* quartett). *Bottom right* quartett of frames shows diffuse late hyperfluorescence and hyperfluorescent inflamed disc

**Fig. 5:**
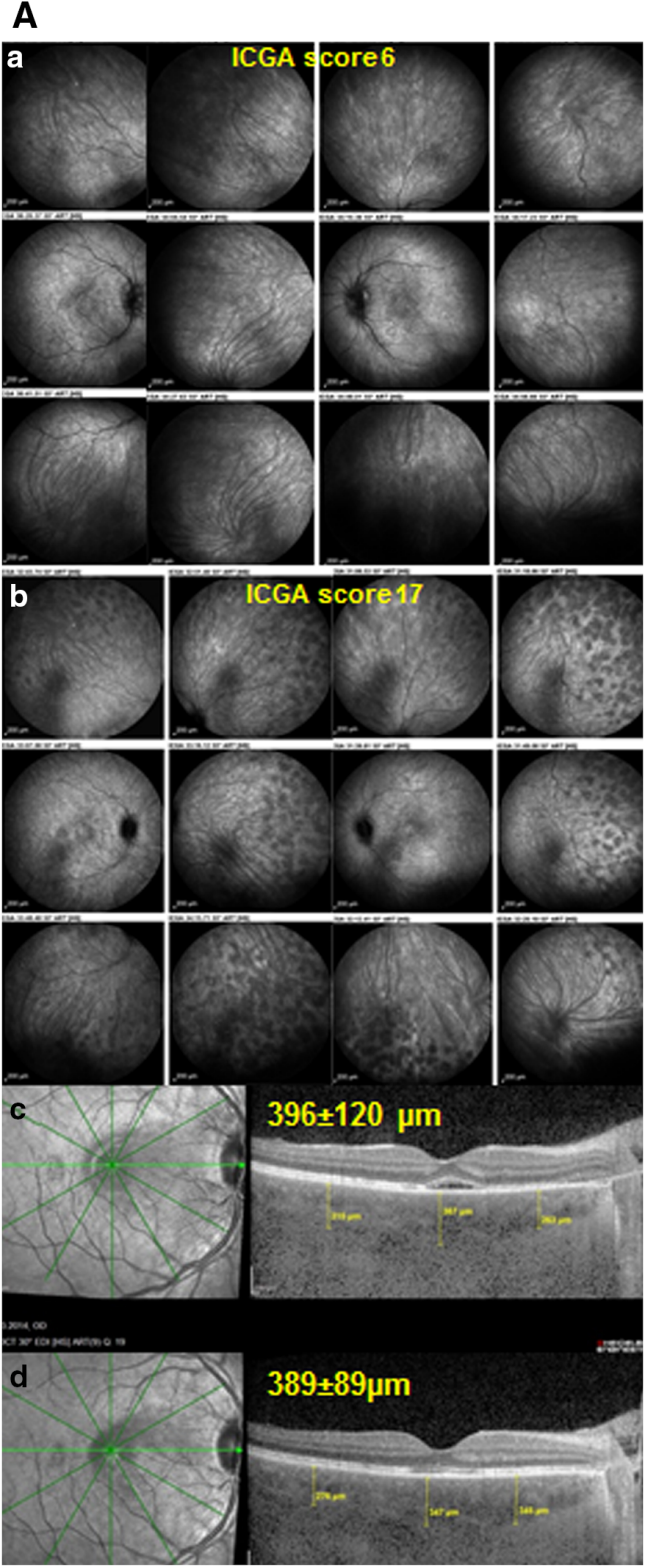

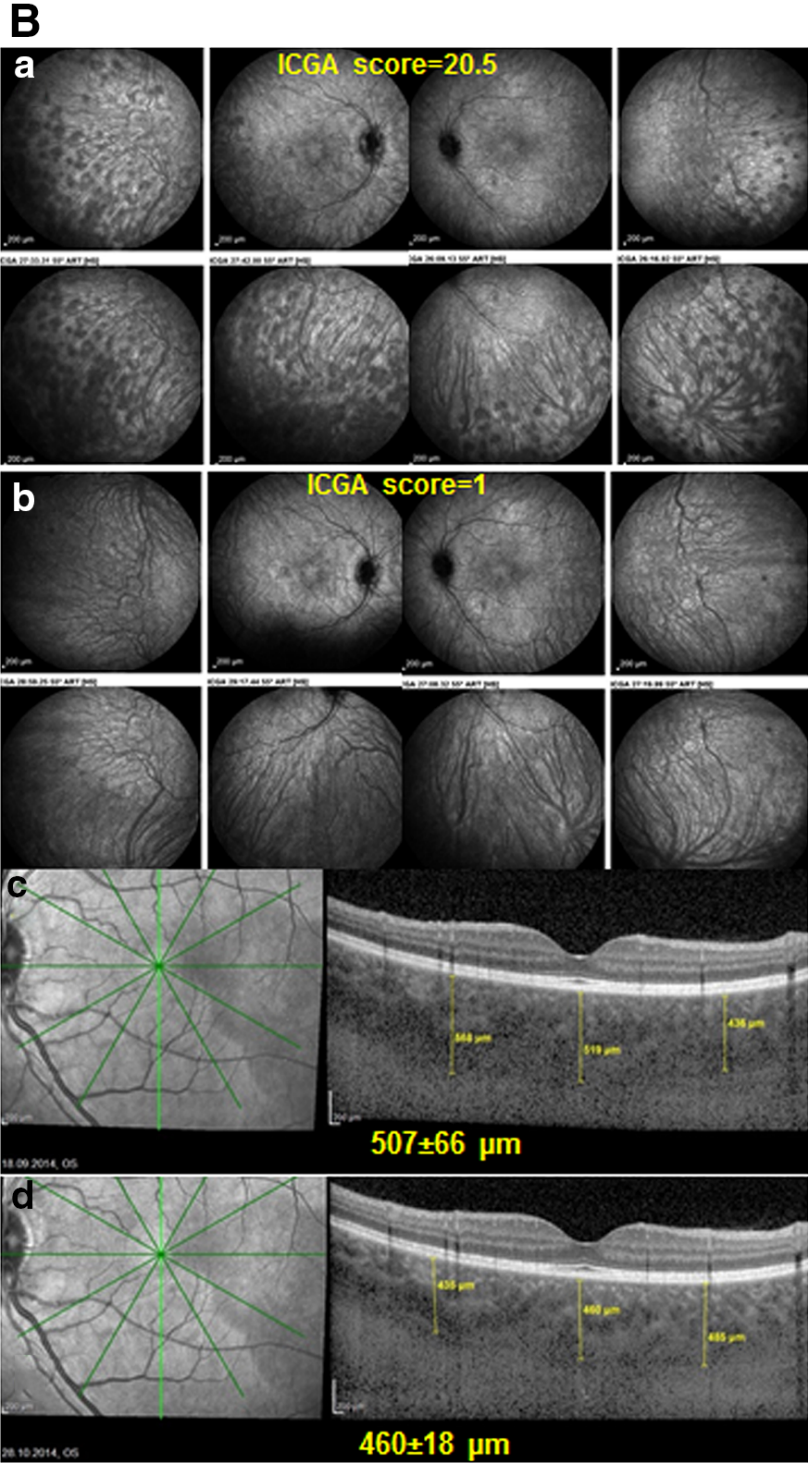
**A** ICGA is more reactive than EDI-OCT for close, visit by visit follow-up. Patient with initial onset VKH disease having responded to initial high-dose IV and then oral corticosteroids, together with mycophenolic acid Myfortic^®^ and cyclosporine (CsA)with reduction of ICGA score from 31/40 to 6/40 (**a**), using an established dual FA/ICGA scoring system [61, 62]. Under Myfortic^®^ and CsA, ICGA score increased again to 17/40 (**b**), not reflected in EDI-OCT that shows slight decrease of choroidal thickness (**c**, **d**). **B** Indocyanine green angiography is more reactive than EDI-OCT for precise follow-up of VKH disease (same patient as in Fig. 3) Patient with VKH disease under Myfortic® and CsA to which choroiditis is not responding with an ICGA score of 20.5/40 (**a**). After 6 months, it was decided to add Remicade® (Infliximab, an anti-TNF-blocker, 5mg/kg per infusion). 5 weeks later (3 infusions), HDDs completely disappeared with a decrease of ICGA score from 20.5 ± 4.9 to 1 (p < 0.03) (**b**), while choroidal thickness showed a slight non-significant difference, 507 ± 66 versus 460 ± 18 μm (p = 0.31) (**c**, **d**)

ICGA is therefore essential in the investigation and follow-up of VKH disease as it brings additional global panfundal information on the crucial, core structure of the disease process, that is not available otherwise. Depth-enhanced choroidal optical coherence tomography of the choroid (EDI-OCT), which shows choroidal thickening, is not capable of providing reliable short-term monitoring regarding the full inflammatory control of the disease in the choroid to adjust therapy, which can be achieved with ICGA [26]. (Fig. 3) However, OCT measurement of choroidal thickness is a good parameter to follow choroidal disease in the long-term [27].

Classical ICGA signs in VKH disease have now been well established. In a Caucasian group of patients, 4 signs consistently found in acute VKH disease have been identified, the most important one being hypofluorescent dark dots (HDDs) indicating stromal foci (granulomas) [22]. (Fig. 4) The three other ICGA signs consistently found and useful for the evaluation and follow-up of choroiditis were early hyperfluorescent choroidal vessels, fuzzy indistinct large choroidal vessels indicating choroidal vasculitis and ICGA disc hyperfluorescence [22]. (Fig. 4) A further study showed that these ICGA signs were also present in Japanese patients and a recent study showed the same in Saudi Arabian patients, indicating that these are universally found signs [28, 29].

ICGA signs were seen in 100% of initial-onset, untreated disease and the first sign to respond to therapy was disc hyperfluorescence [22, 24]. In acute recurrent attacks during the chronic stage of disease, the same signs can be observed. However, in chronic smouldering disease without acute recurrent attacks, only hypofluorescent dark dots (HDDs) and fuzzy indistinct choroidal vessels indicate ongoing choroidal inflammation [22].

Furthermore, it was shown that ICGA was most appropriate for close follow-up of VKH disease, promptly showing subclinical and occult choroidal recurrence of the disease [24]. It also seems to be the most reactive monitoring parameter compared to choroidal OCT less reliable for close follow-up as illustrated in the case shown on Fig. 5. Heidelberg enhanced depth imaging optical coherence tomography (EDI-OCT) or other choroidal OCTs have clearly shown choroidal thickening in VKH disease and progressive decrease of choroidal thickness during treatment and on long-term follow-up [27, 30]. Although thickness is proportional to treatment course and its linear decrease can be clearly documented by choroidal OCT, these changes occur more slowly over time and are less reactive and sensitive to be relied upon for close follow-up [31].

Based on the evidence presented, the second element to be taken into account for optimal management of VKH disease is that ICGA monitoring is essential for precise follow-up of the disease and to verify the absence of occult choroiditis.

## Rationale for improved management of VKH disease

The principles of therapy of VKH disease are to suppress the initial intraocular inflammation in the acute posterior uveitic/exudative stage with early and aggressive use of systemic corticosteroids followed by slow tapering. Such treatment may shorten the duration of the disease, may prevent progression into the chronic stage, and may also reduce the incidence of extraocular manifestations [1]. The intraocular inflammation will proceed to recurrent granulomatous anterior uveitis with typical SGF if not properly treated [1, 9, 10]. Despite proper treatment with corticosteroid monotherapy, several studies reported the development of chronic smouldering and/or recurrent granulomatous inflammation and sunset glow fundus (SGF) with peripapillary atrophy and depigmented small atrophic lesions at the level of retinal pigment epithelium [10, 32].

Recently, Sakata et al. reported a high rate of clinical recurrence in VKH patients treated with early high-dose corticosteroids given within 30 days from disease onset and slow taper, as 79% of patients progressed to chronic recurrent disease and 38% developed subretinal fibrosis [33]. Similarly, Chee et al. observed that one-third of patients receiving high-dose corticosteroid therapy within 2 weeks of onset progressed to chronic recurrent disease [34]. Keino et al. demonstrated that despite high-dose corticosteroid therapy at initial onset, 17.5% of patients developed chronic ocular inflammation [35]. Chronic recurrent VKH disease is significantly associated with more severe anterior segment inflammation at presentation and a worse visual acuity [10] and a worse mean retinal sensitivity [36] compared with initial-onset acute VKH disease receiving appropriate therapy [10]. Studies using laser flare cell photometry demonstrated that both aqueous flare values and cell counts were significantly higher in patients with chronic recurrent VKH disease than those with initial-onset acute VKH disease [37]. Furthermore, chronic recurrent granulomatous inflammation in the anterior segment was more refractory to treatment [36]. Consequently, complications are more common in patients with chronic recurrent VKH disease who present recurrent granulomatous anterior uveitis and SGF [9, 10].

The poor visual prognosis associated with chronic ocular inflammation and the well-documented ocular and systemic complications of long-term high-dose corticosteroid use have led many experts to suggest initiating first-line non-steroidal immunosuppressive therapy using cyclosporine, azathioprine, methotrexate, mycophenolate mofetil or other, combined with systemic corticosteroids early in the course of care of patients to achieve better control of the uveitis and to facilitate earlier tapering of corticosteroids [38–40].

Standard treatment regimens recommend high-dose systemic corticosteroid therapy, with or without intravenous administration during the first 3 days of treatment, tapered over a period of 6–9 months [1, 41–44]. While there is a universal consensus on the treatment of the acute phase of VKH disease where high-dose corticosteroids are necessary to quickly bring inflammation under control, this is not the case for the post-acute phase of the disease [45].

It has now been clearly shown by ICGA monitoring of the choroid during tapering of corticosteroids that active choroidal inflammation can be identified in an apparently quiescent eye explaining the chronic evolution [23–26, 45]. This shows that the standard recommended therapy is indeed suppressing clinically apparent disease but is insufficient to suppress choroidal ICGA detected inflammation [45]. This has led some experts to maintain sufficient therapy including first-line non-steroidal immunosuppressive drugs in the subacute phase and during the tapering process in order to eradicate choroidal inflammation [24, 38–40].

Following the administration of corticosteroid monotherapy tapered over 6–9 months, except for the rare cases that respond to this simple and relatively short corticosteroid therapy classically applied, evolution is unfortunately mostly unfavourable [33]. Even if early and high doses are given, evolution is characterized by chronic smouldering and/or recurrent disease, the third, chronic, stage of the disease, after the prodromal stage and the uveitic/exudative stage [33].

In smouldering disease, subclinical evolution of choroidal inflammation has been clearly documented with the help of ICGA [22–24, 45], explaining the development of SGF despite (usually suboptimal) therapy, resulting from the progressive loss of stromal melanocytes because of an ongoing insufficiently controlled immunological process [33, 45, 46]. Further evidence for the presence of smouldering choroiditis is the low grade intraocular inflammation found by laser flare photometry in a seemingly quiet eye [37].

As the disease process begins to wane, the posterior portion of the globe characteristically shows depigmentation (Fig. 4). This SGF, reflecting the stromal pigment loss, has been described to reach high proportions in most studies and is said to be more pronounced in Asian patients probably because their choroid is more heavily pigmented [35, 47, 48].

Keino et al. followed 102 patients with VKH disease from initial-onset who were treated with high-dose corticosteroid therapy. SGF developed in 67.6% of patients. The mean duration until appearance of SGF was 4.2 ± 2.7 months [49]. Similarly, Lai et al. demonstrated the development of SGF in 51.4% of patients who received oral corticosteroids during the first attack of VKH disease [43]. Several studies reported the significant association between the incidence of chronic ocular inflammation and the development of SGF [10, 35, 50].

SGF is today still presented as the natural course of the disease which was indeed the case at the time when no treatment was available, as described by Koyanagi in the first large series published [51]. To date with the availability of corticosteroids and immunosuppressive drugs, SGF is the result of insufficient treatment that is unable to stop choroidal inflammation but should not be considered any more as the unavoidable evolution of VKH disease [52].

**Fig. 6:**
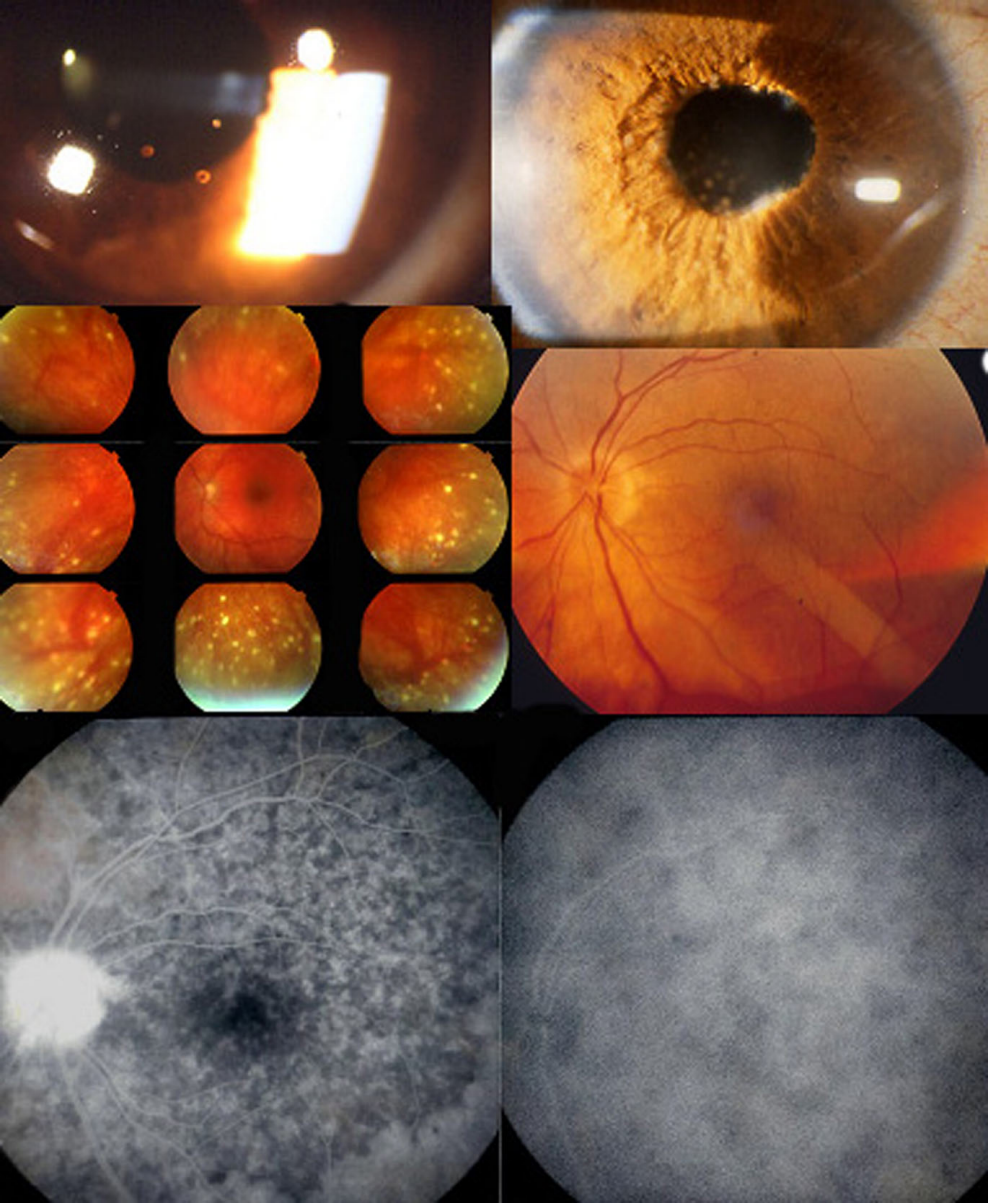
Signs found in chronically evolving disease. Chronic granulomatous uveitis with old pigmented KPs (*top left*), Koeppe nodules, iris infiltration and Busacca nodules (*top right*). Mid-periphery hypopigmented lesions are shown on the *middle left* picture and sunset-glow-fundus on the *right middle* picture. Mottled irregular, disturbed RPE in the posterior pole and high-water marks indicating limit of reattached serous retinal detachment as well as disc hyperfluorescence seen on the FA frame (*bottom left*). Only ICGA can show that disease is still active as evidenced by the numerous dark dots (*bottom right*)

Chronic disease can also present with recurrent acute inflammatory attacks complicating the smouldering evolution, a proof that there are glowing embers in the choroid ready to flare up again [23–25, 47]. These acute recurrent attacks resemble the acute de novo, initial disease with some differences. The characteristics of the recurrence will depend on how important the loss of stromal choroidal melanocytes was during the initial acute attack of the disease and the subsequent smouldering phase of disease. If the loss of melanocytes was important, naturally the preponderant part of the recurrent inflammation will be anterior as it is classically described because this is where the bulk of remaining melanocytes is [9]. However if the loss of choroidal stromal melanocytes was scarce, recurrences can present themselves again as predominantly posterior, which is usually less pronounced than in de novo-initial disease. Anterior granulomatous uveitis is a more prominent feature in depigmented fundi with its classical signs of granulomatous KPs, often pigmented, that can even take a mutton-fat aspect, Koeppe fluffy nodules, diffuse iris infiltration or Busacca nodules in the iris and posterior synechiae [10] (Fig. 6). Sometimes, what seems to be a purely anterior recurrence is associated with a subclinical posterior recurrence only shown by resurgence of hypofluorescent dark dots (HDDs) on ICGA [24].

The high proportion of cases with chronic evolution even with early high-dose corticosteroid therapy should lead the clinician to the conclusion that the present standard treatment relying principally on corticosteroids tapered during 6–9 months is insufficient and represents the third main consideration in the design of an optimized management of VKH disease [33].

## Proposals for an optimized management of VKH disease

The following elements should be considered when designing an improved management for VKH disease: (1) VKH is a primary stromal choroiditis where inflammation is exclusively originating from the choroid, meaning that as soon as choroiditis is eradicated intraocular inflammation is over; (2) henceforth the principal parameter for disease monitoring is ICGA; (3) the presently recommended standard therapy for VKH disease, based on systemic corticosteroids given for 6–9 months, is insufficient and treatment should be stepped-up [38].

We know by now that, when clinically apparent disease is brought under control, this is not sufficient in most cases. ICGA monitoring of VKH patients under treatment has shown that once clinical signs and functional parameters including fundus picture, OCT, FA and visual acuity have normalized, choroidal inflammation is still present and evolving as evidenced by ICGA [22–24, 47]. The persistence of choroidal subclinical disease explains the evolution towards SGF in most cases receiving standard therapy [35, 48, 49].

Taking into account these elements, therapy of VKH disease should have two main phases including (1) treatment of the early uveitic/exudative acute stage of the disease and (2) maintaining sufficient therapy including first-line non-steroidal immunosuppressive drugs long enough leading to resolution of choroidal inflammation, if possible using ICGA assisted tapering of therapy.

### Treatment of the early acute phase of disease

The severity of acute VKH disease needs high-dose corticosteroids given as early as possible [53]. A 3 days course of intravenous methyl-prednisolone (500–1000 mg per day) is recommended, followed by high-dose oral prednisone (1.0–1.2 mg/kg) for 4–6 weeks. Although the need for intravenous corticosteroid administration during the 3 first days of treatment has not really been proven, common sense has it that, in case of hyperacute uveitis, rapid resolution of inflammation is desirable [54].

### Subacute phase of therapy, corticosteroid tapering & discontinuation of therapy

It was shown, using ICGA-monitoring of choroiditis, that in 7/9 patients an immunosuppressive agent had to be added because of recurrent choroidal disease seen during ICGA follow-up [24]. It therefore makes sense to add a non-steroidal immunosuppressant directly at the start of disease with the risk of overtreating some patients that would have responded to corticosteroids alone. Early introduction of non-steroidal immunosuppressive therapy is also indicated because the same study showed that the mean duration of therapy needed for choroiditis-free resolution of disease was 27 months, a duration precluding the use of corticosteroids alone [24]. In case of resolution of choroidal, ICGA monitored, inflammation, tapering down of prednisone to low levels (5–10 mg daily) should be tried within a period of ±4 months, a time-span needed for some immunosuppressants like azathioprine or mycophenolate to reach full activity [38]. An immunosuppressant increasingly chosen by clinicians is mycophenolate mofetil (CellCept^®^, 1000 mg twice daily) or mycophenolic acid (Myfortic^®^, 720 mg twice daily) because this agent is both very effective and usually well tolerated. Other immunosuppressing drugs or biologic agents that have been reported are cyclosporine, anti-TNF agents or interferon-alpha, noting for the latter that it can itself induce VKH disease [55, 56].

Abu El-Asrar et al. evaluated the effectiveness of mycophenolate mofetil as first-line therapy combined with systemic corticosteroids in initial-onset acute uveitis associated with VKH disease [38]. They compared the outcomes in this group with those of another group of patients with acute uveitis associated with VKH disease who were treated with corticosteroid monotherapy or with delayed addition of immunomodulatory therapy. The outcome of this group was previously reported [32]. They demonstrated that the use of mycophenolate mofetil as first-line therapy combined with systemic corticosteroids was safe and effective in the treatment of acute uveitis associated with VKH disease. It has marked corticosteroid-sparing effect and significantly reduced the development of chronic recurrent inflammation and late complications. In addition, mycophenolate mofetil significantly improved visual outcome. None of the patients developed SGF, suggesting that it was effective in controlling progressive subclinical choroidal inflammation. Furthermore, mycophenolate mofetil was effective in preventing the development of vitiligo, poliosis, alopecia, and sensory hearing loss [38].

Some authors proposed ICGA-monitoring of regression of choroidal disease in the convalescent phase of disease and long-term tapering of inflammation suppressive therapy until absence of recurrence of choroidal disease (zero tolerance of choroiditis). Meaningful ICGA-monitoring presupposes an ICGA every 5–6 weeks during the first 4–5 months and then every 2–3 months during the long-term tapering period but there are cost considerations [24]. Performing an ICGA every 6 months in the follow-up of VKH disease cannot be called ICGA-monitoring but represents merely ICGA documented follow-up as the low frequency of ICGAs performed in this study did not allow to modify treatment in a timely fashion [57]. During the tapering phase of corticosteroids and in the convalescent phase when corticosteroids have been stopped, in case of ICGA detected recurrence of subclinical choroidal disease, corticosteroids (oral prednisone) should be re-increased or introduced again to 1 mg/kg for a few days followed by a new taper. Sneaking out with treatment takes much longer than the treatment durations reported in textbooks or in published literature, until successful tapering without subclinical disease recurrence in the choroid is achieved. In a series of 9 patients treated with ICGA-assisted management, the mean duration of treatment was 27.3 months ±38.2 months (range 9–114) which is much longer than the 6 to 9 months cited in the literature [24]. The advantage of such a relentless therapy is a high proportion (7/9 cases, 78%) of ″healed″ cases with no recurrent activity within a mean follow-up period without therapy of 26 ± 14.8 months as well as a low proportion of cases with sunset glow fundus (3/9, 33%) [24]. These results indicate that early and sustained, ICGA-monitored treatment is able to modify the phenotype of VKH disease. A similar achievement with sustained and prolonged immunosuppressive therapy was achieved in birdshot retinochoroiditis, another stromal choroiditis where the development of oval depigmented birdshot lesions could be totally suppressed and the phenotype of the disease was changed by radical therapy [58].

Some authors, in stromal choroiditis, increasingly use early high-dose systemic steroids at start associated with a quickly acting immunosuppressive drug such as cyclosporine together with a well-tolerated immunosuppressant such as mycophenolic acid which needs several months to be fully active [59]. This allows more rapid tapering of corticosteroids and a limited period (4–5 months) of cyclosporine use.

At this point, further studies are looking into the need for non-steroidal immunosuppression for initial-onset disease. In Brazil, a study on nine patients with initial-onset disease treated with highly dosed corticosteroids showed that all presented recurrence of inflammation after 6 months after onset of disease, either detected clinically or subclinically, in a median time of seven months or when the tapering dose of oral corticosteroids was reduced to 0.3 mg/kg/day. It seems that, considering this prospective study, even treatment with highly dosed corticosteroids with a really slow tapering was insufficient to prevent further recurrences and the protocol was changed to early non-steroidal immunosuppression [60]. In Japan, at present, two multicentre trials are being conducted to assess the effect of early non-steroidal immunosuppressive therapy in addition to corticosteroids in initial-onset acute VKH disease. Together with the evidence presented here, this shows that there is a clear trend towards the association of non-steroidal immunosuppressive therapy and corticosteroids in the management of initial-onset VKH disease.

## Perspective and concluding remarks: sunset-glow fundus is no more a fatality

VKH disease is a primary stromal choroiditis, meaning that inflammation takes its origin exclusively in the choroid, reaching other structures only when inflammation spills over to these compartments. Unlike in birdshot retinochoroiditis, where inflammation starts independently in the choroid and at the level of the retina, in VKH disease, inflammation initiates nowhere else than in the choroid which is the structure that should be targeted by therapy. The choroidal compartment, unlike the retina, is easily accessible to systemic therapy. This is the reason why there is increasing evidence that, if treatment is conducted consequently with a zero tolerance to recurrence of choroiditis that can be detected by ICGA or choroidal OCT, a substantial proportion of cases can be healed before depigmentation occurs, meaning that sunset-glow fundus should no more be an inevitable outcome.

## References

[1] Moorthy RS, Inomata H, Rao NA (1995). Vogt–Koyanagi–Harada syndrome. Surv Ophthalmol.

[2] Gocho K, Kondo I, Yamaki K (2001). Identification of autoreactive T cells in Vogt–Koyanagi–Harada disease. Investig Ophthalmol Vis Sci.

[3] Damico FM, Cunha-Neto E, Goldberg AC, Iwai LK, Hammer J, Kalil J, Yamamoto JH (2005). Vogt T-cell recognition and cytokine profile induced by melanocyte epitopes in patients with HLA-DRB1*0405-positive and -negative Vogt–Koyanagi–Harada uveitis. Investig Ophthalmol Vis Sci.

[4] Sugita S, Takase H, Taguchi C, Imai Y, Kamoi K, Kawaguchi T, Sugamoto Y, Futagami Y, Itoh K, Mochizuki M (2006). Ocular infiltrating CD4+ T cells from patients with Vogt-Koyanagi-Harada disease recognize human melanocyte antigens. Investig Ophthalmol Vis Sci.

[5] Abu El-Asrar AM, Struyf S, Kangave D (2011). Cytokine profiles in aqueous humor of patients with different clinical entities of endogenous uveitis. Clin Immunol.

[6] Nakamura S, Nakazawa M, Yoshioka M, Nagano I, Nakamura H, Onodera J, Tamai M (1996). Melanin-laden macrophages in cerebrospinal fluid in Vogt–Koyanagi–Harada syndrome. Arch Ophthalmol.

[7] Noguchi Y, Nishio A, Takase H, Miyanaga M, Takahashi H, Mochizuki M, Kitamura K (2014). Audiovestibular findings in patients with Vogt–Koyanagi–Harada disease. Acta Otolaryngol.

[8] Fang W, Yang P (2008). Vogt–Koyanagi–Harada syndrome. Curr Eye Res.

[9] Yang P, Ren Y, Li B, Fang W, Meng Q, Kijlstra A (2007). Clinical characteristics of Vogt–Koyanagi–Harada syndrome in Chinese patients. Ophthalmology.

[10] Abu El-Asrar AM, Al Tamimi M, Hemachandran S, Al-Mezaine HS, Al-Muammar A, Kangave D (2013). Prognostic factors for clinical outcomes in patients with Vogt–Koyanagi–Harada disease treated with high-dose corticosteroids. Acta Ophthalmol.

[11] Sukavatcharin S, Tsai JH, Rao NA (2007). Vogt–Koyanagi–Harada disease in Hispanic patients. Int Ophthalmol.

[12] Khairallah M (2014). Headache as an initial manifestation of Vogt–Koyanagi–Harada disease. Saudi J Ophthalmol.

[13] Kosma KK, Kararizou E, Markou I, Eforakopoulou E, Kararizos G, Mitsonis C, Gkiatas K (2008). Headache as a first manifestation of Vogt–Koyanagi–Harada disease. Med Princ Pract.

[14] Bouchenaki N, Herbort CP (2001). The contribution of indocyanine green angiography to the appraisal and management of Vogt–Koyanagi–Harada disease. Ophthalmology.

[15] Mantovaqni A, Resta A, Herbort CP, Abu El Asrar A, Kawaguchi T, Mochizuki M, Okada AA, Rao N (2007). Work-up, diagnosis and management of acute Vogt–Koyanagi–Harada disease: a case of acute myopization with granulomatous uveitis. Int Ophthalmol.

[16] Bouchenaki N, Herbort CP, Pleyer U, Mondino B (2004). Stromal choroiditis. Essentials in ophthalmology: uveitis and immunological disorders.

[17] Bouchenaki N, Cimino L, Auer C, Tran VT, Herbort CP (2002). Assessment and classification of choroidal vasculitis in posterior uveitis using indocyanine green angiography. Klin Monbl Augenheilkd.

[18] Papadia M, Herbort CP, Mochizuki M (2010). Diagnosis of ocular sarcoidosis. Ocul Immunol Inflamm.

[19] Papadia M, Herbort CP (2013). Reappraisal of birdshot retinochoroiditis (BRC): a global approach. Graefe’s Arch Clin Exp Ophthalmol.

[20] Khalifa Y, Loh AR, Acharya NR (2009). Fluocinolone acetonide intravitreal implants in Vogt–Koyanagi–Harada disease. Ocul Immunol Inflamm.

[21] Latronico ME, Rigante D, Caso F, Cantarini L, Costa L, Nieves-Martin L, Traversi C, Franceschini R (2015). Bilateral dexamethasone intravitreal implant in a young patient with Vogt–Koyanagi–Harada disease and refractory uveitis. Clin Rheumatol.

[22] Herbort CP, Mantovani A, Bouchenaki N (2007). Indocyanine green angiography in Vogt–Koyanagi–Harada disease: angiographic signs and utility in patient follow-up. Int Ophthalmol.

[23] Da Silva FT, Hirata CE, Sakata VM, Olivalves E, Preti R, Pimentel SL, Gomes A, Takahashi WY, Costa RA, Yamamoto JH (2012). Indocyanine green angiography findings in patients with long-standing Vogt–Koyanagi–Harada disease: a cross-sectional study. BMC Ophthalmol.

[24] Bouchenaki N, Herbort CP (2011). Indocyanine green angiography guided management of Vogt–Koyanagi–Harada disease. J Ophthalmic Vis Res.

[25] Bascal K, Wen DS, Chee SP (2008). Concomitant choroidal inflammation during anterior segment recurrence in Vogt–Koyanagi–Harada disease. Am J Ophthalmol.

[26] Balci O, Gasc A, Jeannin B, Herbort CP (2016). Enhanced depth imaging is less suited than indocyanine green angiography for close monitoring of primary stromal choroiditis: a pilot study. Int Ophthalmol.

[27] Miyanaga M, Kawaguchi T, Miyata K, Horie S, Mochizuki M, Herbort CP (2010). Indocyanine gree angiography findings in initial acute pre-treatment Vogt–Koyanagi–Harada disease in Japanese patients. Jpn J Ophthalmol.

[28] Abouammoh MA, Gupta V, Hemachandran S, Herbort CP, Abu El-Asrar AM (2016). Indocyanine green angiography in initial-onset acute Vogt–Koyanagi–Harada disease. Acta Ophthalmol.

[29] Nakai K, Gomi F, Ikuno Y, Yasuno Y, Nouchi T, Ohguro N, Nishida K (2012). Choroidal observation in Vogt–Koyanagi–Harada disease using high-penetration optical coherence tomography. Graefe’s Arch Clin Exp Ophthalmol.

[30] Da Silva FT, Sakata VM, Nakashima A, Hirata CE, Olivalves E, Takahashi WY, Costa RA, Yamamoto JH (2013). Enhanced depth imaging optical coherence in long-standing Vogt–Koyanagi–Harada disease. Br J Ophthalmol.

[31] Maruko I, Iida T, Sugano Y, Oyamada H, Sekiryu T, Fujiwara T, Spaide RF (2011). Subfoveal choroidal thickness after treatment of Vogt–Koyanagi–Harada disease. Retina.

[32] Al-Kharashi AS, Aldibhi H, AL-Fraykh H, Kangave D, Abu El-Asrar AM (2007). Prognostic factors in Vogt–Koyanagi–Harada disease. Int Ophthalmol.

[33] Sakata VM, da Silva FT, Hirata CE, Marin ML, Rodrigues H, Kalil J, Costa RA, Yamamoto JH (2015). High-rate of clinical recurrence in patients with Vogt-Koyanagi-Harada disease treated with early high-dose corticosteroids. Graefes Arch Clin Exp Ophthalmol.

[34] Chee SP, Jap A, Bascal K (2007). Spectrum of Vogt–Koyanagi–Harada disease in Singapore. Int Ophthalmol.

[35] Keino H, Goto H, Usui M (2002). Sunset glow fundus in Vogt–Koyanagi–Harada disease with or without chronic ocular inflammation. Graefe’s Arch Clin Exp Ophthalmol.

[36] Abu El-Asrar AM, Al Mudhaiyan T, Al Najashi AA, Hemachandran S, Hariz R, Mousa A, Al-Muammar A (2016). Chronic recurrent Vogt-Koyanagi-Harada disease and development of “sunset glow fundus” predict a worse retinal sensitivity. Ocul Immunol Inflamm.

[37] Fang W, Zhou H, Yang P, Huang X, Wang L, Kijlstra A (2008). Longitudinal quantification of aqueous flare and cells in Vogt–Koyanagi–Harada disease. Br J Ophthalmol.

[38] Abu El-Asrar AM, Hemachandran S, Al-Mezaine HS, Kangave D, Al-Muammar AM (2012). The outcomes of mycophenolate mofetil therapy combined with systemic corticosteroids in acute uveitis associated with Vogt–Koyanagi–Harada disease. Acta Ophthalmol.

[39] Bouchenaki N, Morisod L, Herbort CP (2000). Vogt–koyanagi–Harada disease: importance of rapid diagnosis and therapeutic intervention. Klin Monbl Augenheilkd.

[40] Paredes I, Ahmed M, Foster CS (2006). Immunomodulatory therapy for Vogt–Koyanagi–Harada patients as first-line therapy. Ocul Immunol Inflamm.

[41] Touitou V, Escande C, Bodaghi B, Cassoux N, Wechsler B, Lemaître C, Tran TH, Fardeau C, Piette JC, LeHoang P (2005). Diagnostic and therapeutic management of Vogt–Koyanagi–Harada syndrome. J Fr Optalmol.

[42] Sheu SJ (2005). Update on uveomeningoencephalitides. Curr Opin Neurol.

[43] Rubsamen PE, Gass JDM (1991). Vogt–Koyanangi–Harada syndrome: clinical course, therapy, and complications. Arch Ophthalmol.

[44] Lai TY, Chan RP, Chan CK, Lam DS (2009). Effects of the duration of initial oral corticosteroid treatment on the recurrence of inflammation in Vogt–Koyanagi–Harada disease. Eye.

[45] Miyanaga M, Kawaguchi T, Shimizu K, Miyata K, Michizuki M (2007). Influence of early cerebrospinal fluid-guided diagnosis and early high-dose corticosteroid therapy on ocular outcomes of Vogt–Koyanagi–Harada disease. Int Ophthalmol.

[46] Damico FM, Bezzera FT, Silva GC, Gasparin F, Yamamoto JH (2007). New insights into Vogt–Koyanagi–Harada disease. Arq Bras Oftalmol.

[47] Kawaguchi T, Horie S, Bouchenaki N, Ohno-Matsui K, Mochizuki M, Herbort CP (2010). Suboptimal therapy controls clinically apparent disease but not subclinical progression of Vogt–Koyanagi–Harada disease. Int Ophthalmol.

[48] Suzuki S (1999). Quantitative evaluation of sunset glow fundus in Vogt–Koyanagi–Harada disease. Jpn J Ophthalmol.

[49] Keino H, Goto H, Mori H, Iwasaki T, Usui M (2006). Association between severity of inflammation in CNS and development of sunset glow fundus in Vogt–Koyanagi–Harada disease. Am J Ophthalmol.

[50] Lee E, Lee S, Yu H (2015). A clinical grading system based on ultra-wide field retinal imaging for sunset glow fundus in Vogt–Koyanagi–Harada disease. Graefes Arch Clin Exp Ophthalmol.

[51] Koyanagi Y (1929). Dysakusis, alopecia und poliosis bei schwerer uveitis nicht traumatischen ursprungs. Klin Monatsbl Augenheilkd.

[52] Rao NA, Gupta A, Dustin L, Chee SP, Okada AA, Khairallah M, Bodaghi B, LeHoang P, Accorinti M, Mochizuki M, Prabriputaloong T, Read RW (2010). Frequency of distinguishing clinical features in Vogt–Koyanagi–Harasa disease. Ophthalmology.

[53] Bouchenaki N, Morisod L, Herbort CP (2000). Vogt–Koyanagi–Harada disease: importance of rapid diagnosis and therapeutic intervention. Klin Monatsbl Augenheilkd.

[54] Read RW, Yu F, Accorinti Bodaghi M, Bodaghi B, Chee SP, Fardeau C, Goto H, Holland GN, Kawashima H, Kojima E, LeHoang P, Lemaitre C, Okada AA, Pivetti-Pezzi P, Secchi A, See RF, Tabbara KF, Usui M, Rao NA (2006). Evaluation of the effect on outcomes of the route of administration of corticosteroids in acute Vogt–Koyanagi–Harada disease. Am J Ophthalmol.

[55] Wang Y, Gaudio PA (2008). Infliximab therapy for 2 patients with Vogt–Koyanagi–Harada syndrome. Ocul Immunol Inflamm.

[56] Touitou V, Sene D, Fardeau C, Boutin THD, Duhaut P, Piette JC, LeHoang P, Cacoub P, Bodaghi B (2007). Interferon-alpha 2a and Vogt–Koyanagi–Harada disease: a double-edged sword. Int Ophthalmol.

[57] Chee SP, Jap A (2013). The outcomes of indocyanine green angiography monitored immunotherapy in Vogt–Koyanagi–Harada disease. Br J Ophthalmol.

[58] Knecht PB, Papadia M, Herbort CP (2014). Early and sustained treatment modifies the phenotype of birdshot retinochoroiditis. Int Ophthalmol.

[59] Arcinue CA, Radwan A, Lebanan MO, Foster CS (2013). Comparison of two different combination immunosuppressive therapies in the treatment of Vogt–Koyanagi–Harada syndrome. Ocul Immunol Inflamm.

[60] Sakata VM et al. (2015) Investig Ophthalmol Vis Sci 56: ARVO E-Abstract 2075

[61] Tugal-Tutkun I, Herbort CP, Khairallah M, Angiography Scoring for Uveitis Working Group (ASUWOG) (2010). Scoring of dual fluorescein and ICG inflammatory angiographic signs for the grading of posterior segment inflammation (dual fluorescein and ICG angiographic scoring system for uveitis). Int Ophthalmol.

[62] Tugal-Tutkun I, Herbort CP, Khairallah M, Mantovani A (2010). Interobserver agreement in scoring of dual fluorescein and ICG inflammatory angiographic signs for the grading pf posterior segment inflammation. Ocul Immunol Inflamm.

